# Risk factors for the development of tinea pedis and onychomycosis: Real‐world evidence from a single‐podiatry center, large‐scale database in Japan

**DOI:** 10.1111/1346-8138.16991

**Published:** 2023-10-30

**Authors:** Takasuke Ogawa, Akinori Matsuda, Yumi Ogawa, Rica Tanaka

**Affiliations:** ^1^ Department of Dermatology and Allergology Juntendo University Graduate School of Medicine Tokyo Japan; ^2^ Podiatry Center Juntendo University Hospital Tokyo Japan; ^3^ Division of Regenerative Therapy Juntendo University Graduate School of Medicine Tokyo Japan; ^4^ Department of Plastic and Reconstructive Surgery Juntendo University School of Medicine Tokyo Japan

**Keywords:** large‐scale database, onychomycosis, real‐world, risk factor, tinea pedis

## Abstract

Dermatomycosis, including tinea pedis and onychomycosis, is frequently encountered in routine medical care in Japan. Identifying the risk factors for tinea pedis and onychomycosis development is important to encourage hospital visits by patients who may have these diseases but who are not undergoing any treatment. This approach may lead to the prevention of disease progression and the spread of infections to others. Risk factors for onychomycosis development have been reported both in and outside of Japan. However, most of the risk factors were identified based on a multicenter, questionnaire survey study and included evidence obtained from unclear or inconsistent diagnostic criteria for tinea pedis, onychomycosis, and identified risk factors. The current study analyzed the risk factors for developing tinea pedis and onychomycosis in real‐world practice in Japan using a single‐center, large‐scale database that included the data of patients managed with consistent diagnostic criteria at the Podiatry Center of Juntendo University Hospital. A total of 2476 patients (1012 males, 1464 females) with a mean age of 63.4 years were included. Among these patients, 337 (13.6%) had tinea pedis and 346 (14.0%) had onychomycosis. A total of 259 patients (~ 75% of each patient population) had both diseases concomitantly. Multivariate logistic regression analysis adjusted for the possible risk factors of age (per 10 years), sex, diabetes, dialysis, visual impairment, ulcer history, lower‐limb ischemia (LLI), and diabetic peripheral neuropathy (DPN) revealed that advanced age, male sex, diabetes, and LLI were independent risk factors for the development of tinea pedis. In addition, DPN was an independent risk factor for developing onychomycosis. We believe that these data are useful for identifying patients who are at high risk of developing tinea pedis and onychomycosis, which may result in disease prevention and suppression in real‐world clinical practice in Japan.

## 1 INTRODUCTION

Dermatomycosis, including tinea pedis and onychomycosis, is common worldwide,^1^, ^2^ and is frequently encountered in routine medical care in Japan.^3^ The prevalence of tinea pedis and onychomycosis in Japan is estimated to be 21.6% (~ 25 million patients) and 10.0% (~ 12 million patients), respectively.^4^

Patients with onychomycosis are not merely concerned about the cosmetic changes caused by the disease as onychomycosis can be painful, significantly affect a patient's quality of life, and lead to serious complications.^2^, ^5^ Patients with onychomycosis may cause the spread of the disease to others; however, many patients remain unmedicated in real‐world care in Japan.^6^ Therefore, it is necessary to encourage hospital visits by unmedicated, potential patients who are at risk of developing tinea pedis and onychomycosis, and to provide these patients with information on the potential hazards of these diseases.

The risk factors for the development of onychomycosis that have been reported outside Japan include male sex, advanced age, family or personal history of onychomycosis, tinea pedis, nail trauma, fomite exposure, diabetes, peripheral vascular disease, immunosuppression, and psoriasis.^5^ Watanabe et al. reported that bone and joint disease, palmoplantar hyperhidrosis, sports activity, and climate are associated with onychomycosis development, according to a random sampling survey of dermatology outpatients in 1999 and 2000, particularly those with tinea pedis and onychomycosis.^7^ Most studies that have analyzed the risk factors for these diseases were based on multicenter, questionnaire surveys and included evidence obtained from unclear or inconsistent diagnostic criteria for diseases such as tinea pedis, onychomycosis, and the identified risk factors. In the current study, risk factors for the development of tinea pedis and onychomycosis in real‐world practice in Japan were analyzed using a single‐center, large‐scale database that includes the data of patients managed with consistent diagnostic criteria at the Podiatry Center of Juntendo University Hospital.

## 2 METHODS

### 2.1 Study design and patients

This study was a single‐center, retrospective, observational study that used a large‐scale database to analyze the risk factors for the development of tinea pedis and onychomycosis in real‐world clinical practice in Japan. Patients aged 20 years or older who visited the Podiatry Center of Juntendo University Hospital between June 2019 and December 2021 were included in the study. Patients who did not agree to participate in the study and those who were deemed inappropriate as study participants by the principal investigator were excluded.

### 2.2 Analysis of the possible risk factors for developing tinea pedis and onychomycosis

Tinea pedis and onychomycosis were diagnosed by direct microscopy using potassium hydroxide staining. The following possible risk factors were recorded: (1) basic information (patient age, sex, body mass index, chief complaint, and diagnosis) and (2) the presence of associated diseases (tinea pedis, onychomycosis, and diabetes) and symptoms (lower‐limb ischemia [LLI], diabetic peripheral neuropathy [DPN], visual impairment, dialysis, and history of leg ulcers). Patients with skin tissue perfusion pressure (SPP) of <80 mmHg were diagnosed with LLI. Using the SPP value in each patient, the severity of LLI was categorized as follows: >80 mmHg, normal; <80–50 mmHg, borderline; <50–40 mmHg, suspected peripheral arterial disease; and < 40 mmHg, difficult wound healing. The presence of DPN in diabetic patients was tested using the DPNCheck™ (NeuroMetrix), which is a point‐of‐care, nerve conduction device that is widely used for the detection and severity evaluation of DPN in Japanese clinical practice.^8^, ^9^, ^10^ DPN severity was categorized as normal, mild, moderate, or severe according to the instruction manual for DPNCheck™. Non‐diabetic patients who did not undergo the DPN test were considered to have no DPN. The presence or absence of visual impairment was determined based on the medical records at the hospital visit.

### 2.3 Infection area in patients with onychomycosis

The clinical photographic data of patients with foot diseases recorded in the database of the Podiatry Center of Juntendo University Hospital were used to analyze the infection area in patients with onychomycosis. The clinical photographic data of the patients at their first visit and at 1 year (closest day to 1 year) after their first visit were analyzed for the infection area using ImageJ software (version. 1.53a/Java 1.8.0_112). If multiple toes were involved in a patient with onychomycosis, only one representative toe was followed up.

With reference to a previous report,^11^ the improvement rate was calculated using the following formula:

(Affected nail area at first visit − Affected nail area at observation)/Affected nail area at first visit × 100.

Improvement degree was classified into five categories: no clearance, 0%–10%; slight clearance, 11%–40%; moderate clearance, 41%–70%; significant clearance, ≥71%; and complete cure, 100% of improvement accompanied by a negative mycological test result from the direct microscopy using potassium hydroxide staining.

### 2.4 Ethical review and informed consent

This study was approved by the institutional review board of Juntendo University (approval number: E21‐0301). Opt‐out informed consent was obtained from this retrospective, medical‐record–based study. Information on the study, including its purpose, was posted on the website of our center to ensure that patients had the opportunity to opt out.

### 2.5 Statistical analysis

#### 2.5.1 Analysis of the risk factors

Univariate and multivariate logistic regression analyses were performed using the presence or absence of tinea pedis and onychomycosis as objective variables. Risk factors for developing tinea pedis or onychomycosis were obtained from medical records as explanatory variables. A multivariate logistic regression analysis was performed using the forced entry method. The results are presented as odds ratios and 95% confidence intervals to identify risk factors. In addition, SPP and DPN were categorized by severity, and the association between severity (per 1 category increase in severity) and the development of tinea pedis or onychomycosis was also examined.

#### 2.5.2 Exploratory analysis for the use‐status of therapeutic medicines for onychomycosis

The data of patients who visited the Podiatry Center of Juntendo University Hospital between June 2019 and December 2020 were analyzed. For patients with onychomycosis, information on therapeutic efficacy and factors influencing efficacy in therapeutic interventions were explored. The differences in the improvement rate and the improvement degree between the three treatment groups (efinaconazole, luliconazole, and fosravuconazole) were examined. To assess the improvement rate, a one‐way ANOVA and Bonferroni correction were used for three‐group and paired comparisons, respectively. To assess the improvement degree, the Kruskal‐Wallis test and Mann–Whitney *U* test with Bonferroni correction were used for the three‐group and paired comparisons, respectively (the improvement degree was treated as an ordinal scale).

## 3 RESULTS

### 3.1 Patient characteristics

A total of 2476 patients (1012 males, 1464 females) were included in this study. The mean age was 63.4 years. The study included 337 patients with tinea pedis (13.6%) and 346 patients with onychomycosis (14.0%). Among these patients, 259 patients (~ 75% of each patient population) had both diseases concomitantly. Table 1 lists the characteristics of all patients and those with tinea pedis and onychomycosis.

**TABLE 1:** Patient characteristics in total patients and patients with tinea pedis and onychomycosis.

Patient background factors	Total	Tinea pedis	Onychomycosis
	*n*	*n* (%)	*n* (%)
Total	2476	337 (13.61)	346 (13.97)
Age (years)			
< 40	250	5 (2.00)	3 (1.20)
40–49	206	21 (10.19)	17 (8.25)
50–59	383	45 (11.75)	43 (11.23)
60–69	528	81(15.34)	84 (15.91)
≥70	1109	185 (16.68)	199 (17.94)
Sex			
Male	1012	196 (19.37)	204 (20.16)
Female	1464	141 (9.63)	142 (9.70)
Diabetes			
Absent	1910	166 (8.69)	147 (7.70)
Present	566	171 (30.21)	199 (35.16)
Dialysis			
Absent	2362	307 (13.00)	306 (12.96)
Present	114	30 (26.32)	40 (35.09)
Diabetes/Dialysis			
Absent/Absent	1877	159 (8.47)	139 (7.41)
Absent/Present	33	7 (21.21)	8 (24.24)
Present/Absent	485	148 (30.52)	167 (34.43)
Present/Present	81	23 (28.40)	32 (39.51)
Visual impairment			
Absent	2339	289 (12.36)	278 (11.89)
Present	137	48 (35.04)	68 (49.64)
Ulcer history			
Absent	2103	248 (11.79)	231 (10.98)
Present	373	89 (23.86)	115 (30.83)
LLI			
Absent	2238	247 (11.04)	220 (9.83)
Present	238	90 (37.82)	126 (52.94)
DPN			
Absent	2202	241 (10.94)	210 (9.54)
Present	274	96 (35.04)	136 (49.64)

Abbreviations: DPN, diabetic peripheral neuropathy; LLI, lower‐limb ischemia.

### 3.2 Investigation of risk factors for developing tinea pedis and onychomycosis

**FIGURE 1:**
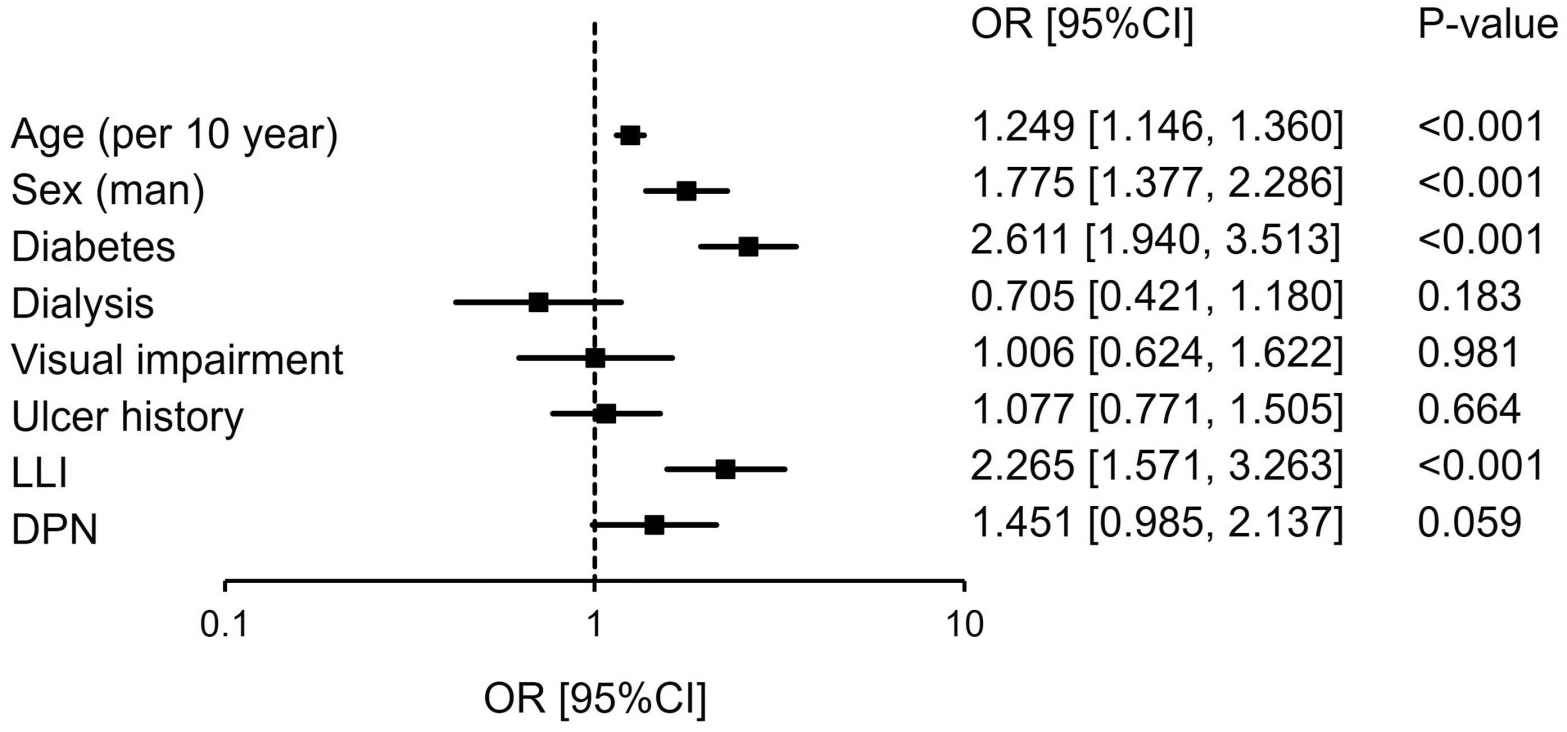
Analysis of the risk factors for developing tinea pedis. Multivariate logistic regression analysis was performed using age (per 10 years), sex, diabetes, dialysis, visual impairment, ulcer history, lower‐limb ischemia (LLI), and diabetic peripheral neuropathy (DPN) as adjustment factors. CI, confidence interval; OR, odds ratio.

**FIGURE 2:**
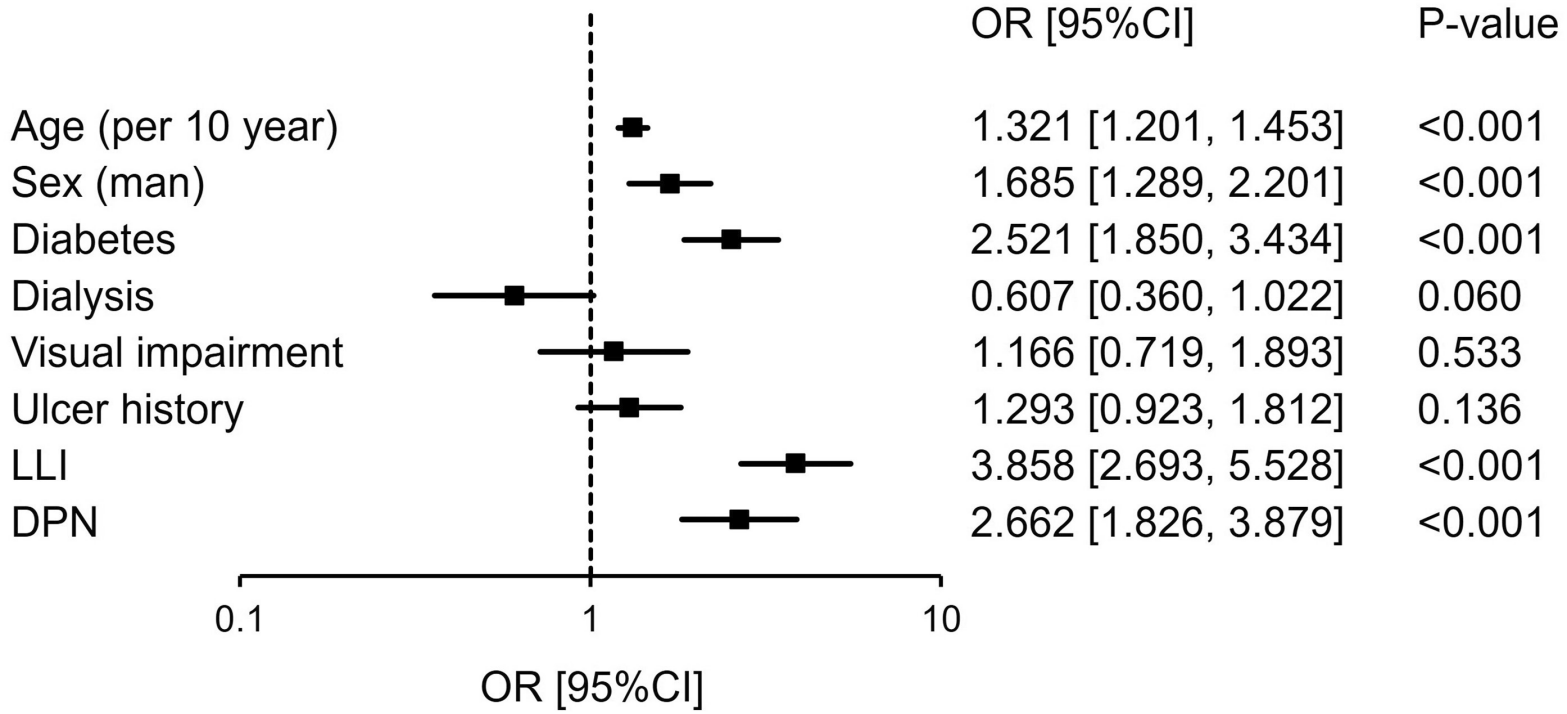
Analysis of the risk factors for developing onychomycosis. Multivariate logistic regression analysis was performed using age (per 10 years), sex, diabetes, dialysis, visual impairment, ulcer history, lower‐limb ischemia (LLI), and diabetic peripheral neuropathy (DPN) as adjustment factors. CI, confidence interval; OR, odds ratio.

Univariate and multivariate logistic regression analyses (forced entry method) were performed to investigate the risk factors for the development of tinea pedis and onychomycosis using the possible risk factors of age (per 10 years), sex, diabetes, dialysis, visual impairment, ulcer history, LLI, and DPN. Multivariate logistic regression analysis was adjusted for all possible risk factors and revealed that advanced age, male sex, diabetes, and LLI were independent risk factors for the development of tinea pedis (Table 2, Figure 1). DPN was also an independent risk factor for developing onychomycosis (Table 3, Figure 2).

**TABLE 2:** Analysis of the risk factors for developing tinea pedis.

Patients background factors	Tinea pedis					
	Absent	Present	Univariate analysis		Multivariate analysis^a^	
	*n* (%)	*n* (%)	OR (95%CI)	*p*‐value	OR (95%CI)	*p*‐value
Total	2139	337				
Age (years)						
<40	245 (11.5)	5 (1.5)	1.000 (Reference)			
40–49	185 (8.6)	21 (6.2)	5.562 (2.059–15.026)	<0.001		
50–59	338 (15.8)	45 (13.4)	6.524 (2.552–16.675)	<0.001		
60–69	447 (20.9)	81 (24.0)	8.879 (3.551–22.203)	<0.001		
≥70	924 (43.2)	185 (54.9)	9.811 (3.991–24.115)	<0.001		
Age (per 10 years)			1.297 (1.199–1.404)	<0.001	1.249 (1.146–1.360)	<0.001
Sex						
Male	816 (38.1)	196 (58.2)	2.254 (1.785–2.846)	0.000	1.775 (1.377–2.286)	<0.001
Female	1323 (61.9)	141 (41.8)	1.000 (Reference)		1.000 (Reference)	
Diabetes						
Absent	1744 (81.5)	166 (49.3)	1.000 (Reference)	<0.001	1.000 (Reference)	<0.001
Present	395 (18.5)	171 (50.7)	4.548 (3.578–5.781)		2.611 (1.940–3.513)	
Dialysis						
Absent	2055 (96.1)	307 (91.1)	1.000 (Reference)	<0.001	1.000 (Reference)	0.183
Present	84 (3.9)	30 (8.9)	2.391 (1.549–3.689)		0.705 (0.421–1.180)	
Diabetes/Dialysis						
Absent/Absent	1718 (80.3)	159 (47.2)	1.000 (Reference)			
Absent/Present	26 (1.2)	7 (2.1)	2.909 (1.243–6.808)	0.014		
Present/Absent	337 (15.8)	148 (43.9)	4.745 (3.686–6.108)	<0.001		
Present/Present	58 (2.7)	23 (6.8)	4.285 (2.574–7.132)	<0.001		
Visual impairment						
Absent	2050 (95.8)	289 (85.8)	1.000 (Reference)	<0.001	1.000 (Reference)	0.981
Present	89 (4.2)	48 (14.2)	3.826 (2.637–5.549)		1.006 (0.624–1.622)	
Ulcer history						
Absent	1855 (86.7)	248 (73.6)	1.000 (Reference)	<0.001	1.000 (Reference)	0.664
Present	284 (13.3)	89 (26.4)	2.344 (1.785–3.078)		1.077 (0.771–1.505)	
LLI						
Absent	1991 (93.1)	247 (73.3)	1.000 (Reference)	<0.001	1.000 (Reference)	<0.001
Present	148 (6.9)	90 (26.7)	4.902 (3.655–6.574)		2.265 (1.571–3.263)	
DPN						
Absent	1961 (91.7)	241 (71.5)	1.000 (Reference)	<0.001	1.000 (Reference)	0.059
Present	178 (8.3)	96 (28.5)	4.388 (3.310–5.818)		1.451 (0.985–2.137)	

Abbreviations: CI, confidence interval; DPN, diabetic peripheral neuropathy; LLI, lower‐limb ischemia; OR, odds ratio.

^a^ Multivariate logistic regression analysis was performed using age (per 10 years), sex, diabetes, dialysis, visual impairment, ulcer history, LLI, and DPN as adjustment factors.

**TABLE 3:** Analysis of the risk factors for developing onychomycosis.

Patients background factors	Onychomycosis					
	Absent	Present	Univariate analysis		Multivariate analysis^a^	
	*n* (%)	*n* (%)	OR (95%CI)	*p*‐value	OR (95% CI)	*p*‐value
Total	2130	346				
Age (years)						
<40	247 (11.6)	3 (0.9)	1.000 (Reference)			
40–49	189 (8.9)	17 (4.9)	7.460 (2.139–25.640)	0.002		
50–59	340 (16.0)	43 (12.4)	10.413 (3.194–33.948)	<0.001		
60–69	444 (20.8)	84 (24.3)	15.557 (4.873–49.792)	<0.001		
≥70	910 (42.7)	199 (57.5)	18.005 (5.708–56.790)	<0.001		
Age (per 10 years)			1.373 (1.265–1.490)	<0.001	1.321 (1.201–1.453)	<0.001
Sex						
Male	808 (37.9)	204 (59.0)	2.351 (1.865–2.962)	<0.001	1.685 (1.289–2.201)	<0.001
Female	1322 (62.1)	142 (41.0)	1.000 (Reference)		1.000 (Reference)	
Diabetes						
Absent	1763 (82.8)	147 (42.5)	1.000 (Reference)	<0.001	1.000 (Reference)	<0.001
Present	367 (17.2)	199 (57.5)	6.503 (5.110–8.275)		2.521 (1.850–3.434)	
Dialysis						
Absent	2056 (96.5)	306 (88.4)	1.000 (Reference)	<0.001	1.000 (Reference)	0.060
Present	74 (3.5)	40 (11.6)	3.632 (2.427–5.434)		0.607 (0.360–1.022)	
Diabetes/Dialysis						
Absent/Absent	1738 (81.6)	139 (40.2)	1.000 (Reference)			
Absent/Present	25 (1.2)	8 (2.3)	4.001 (1.772–9.036)	0.001		
Present/Absent	318 (14.9)	167 (48.3)	6.566 (5.089–8.472)	<0.001		
Present/Present	49 (2.3)	32 (9.2)	8.166 (5.064–13.167)	<0.001		
Visual impairment						
Absent	2061 (96.8)	278 (80.3)	1.000 (Reference)	<0.001	1.000 (Reference)	0.533
Present	69 (3.2)	68 (19.7)	7.306 (5.110–10.447)		1.166 (0.719–1.893)	
Ulcer history						
Absent	1872 (87.9)	231 (66.8)	1.000 (Reference)	<0.001	1.000 (Reference)	0.136
Present	258(12.1)	115 (33.2)	3.612 (2.789–4.679)		1.293 (0.923–1.822)	
LLI						
Absent	2018 (94.7)	220 (63.6)	1.000 (Reference)	<0.001	1.000 (Reference)	<0.001
Present	112 (5.3)	126 (36.4)	10.319 (7.721–13.792)		3.858 (2.693–5.528)	
DPN						
Absent	1992 (93.5)	210 (60.7)	1.000 (Reference)	<0.001	1.000 (Reference)	<0.001
Present	138 (6.5)	136 (39.3)	9.348 (7.092–12.322)		2.662 (1.826–3.879)	

Abbreviations: CI, confidence interval; DPN, diabetic peripheral neuropathy; LLI, lower‐limb ischemia; OR, odds ratio.

^a^ Multivariate logistic regression analysis was performed using age (per 10 years), sex, diabetes, dialysis, visual impairment, ulcer history, LLI, and DPN as adjustment factors.

### 3.3 Association between the development of tinea pedis or onychomycosis with the severity of SPP and DPN

**FIGURE 3:**
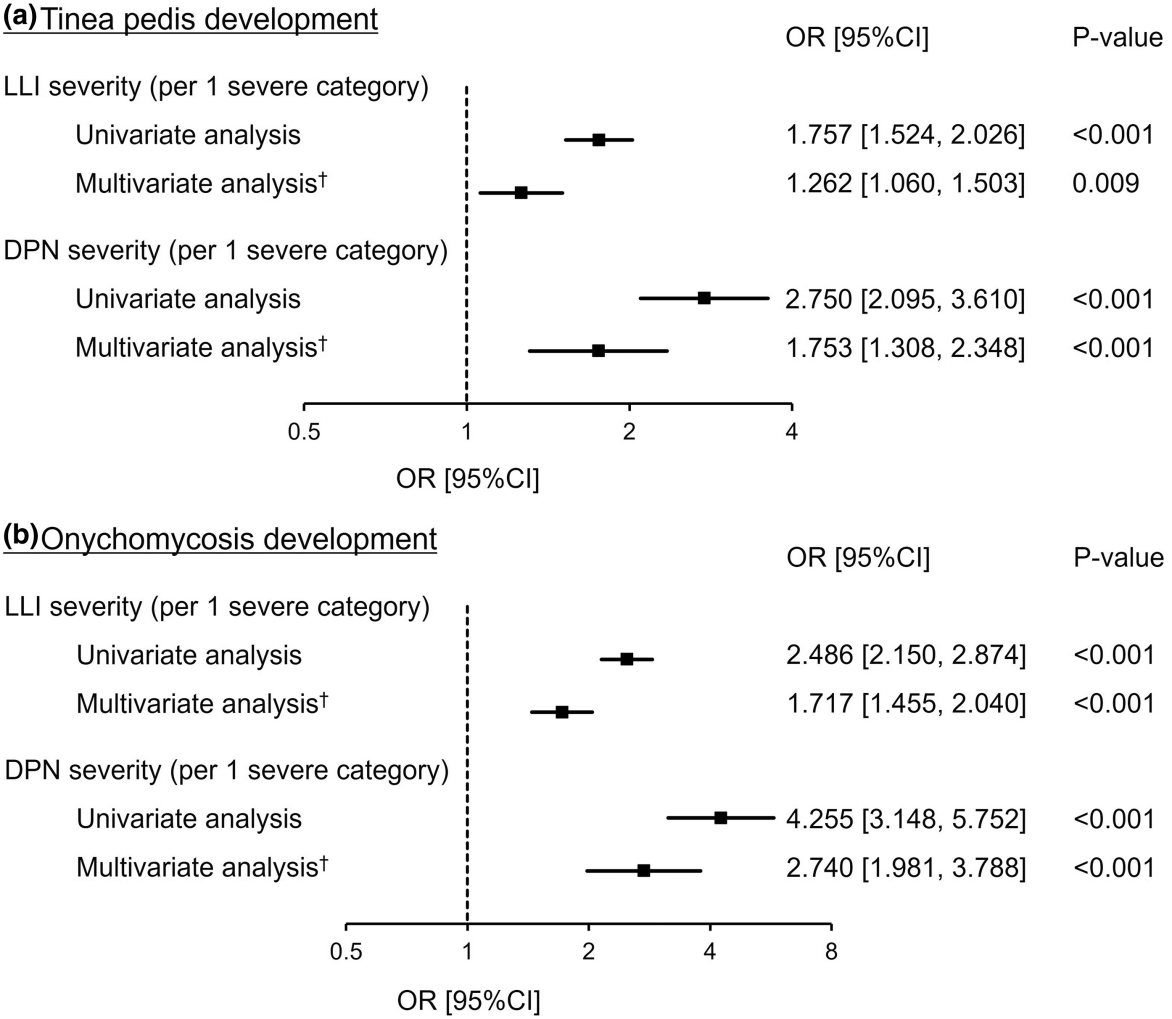
Association of tinea pedis (a) or onychomycosis development (b) with lower‐limb ischemia (LLI) or diabetic peripheral neuropathy (DPN) severity. †Multivariate logistic regression analysis was performed using age, sex, diabetes, dialysis, visual impairment, and ulcer history as adjustment factors. CI, confidence interval; OR, odds ratio.

Univariate and multivariate logistic regression analyses revealed a statistically significant association between the severity of LLI, as assessed using SPP or DPN, and the development of tinea pedis (Figure 3a). These associations were also found between onychomycosis severity and development (Figure 3b).

### 3.4 Exploratory analysis: use‐status of therapeutic medicines for onychomycosis and the clinical effects of these medicines

The use‐status of therapeutic medicines for onychomycosis was investigated in the 261 patients with onychomycosis who visited the Podiatry Center of Juntendo University Hospital between June 2019 and December 2020. The breakdown was as follows: efinaconazole, 149 patients; luliconazole, 50 patients; fosravuconazole, 31 patients; and others, 31 patients (no treatment, eight patients; topical agents without indication for onychomycosis, seven patients; terbinafine, two patients; and multiple drugs, 14 patients). Among the 230 patients treated with efinaconazole, luliconazole, and fosravuconazole, clinical photographic data for analyzing the infection areas both at the first visit and at the evaluation (1 year after the first visit) were available for 89 patients (55 patients treated with efinaconazole, 10 patients treated with luliconazole, and 24 patients treated with fosravuconazole). The dropout rate at 1 year after the first visit was 63.1% (94/149 patients) for efinaconazole, 80.0% (40/50 patients) for luliconazole, and 22.6% (7/31 patients) for fosravuconazole.

**FIGURE 4:**
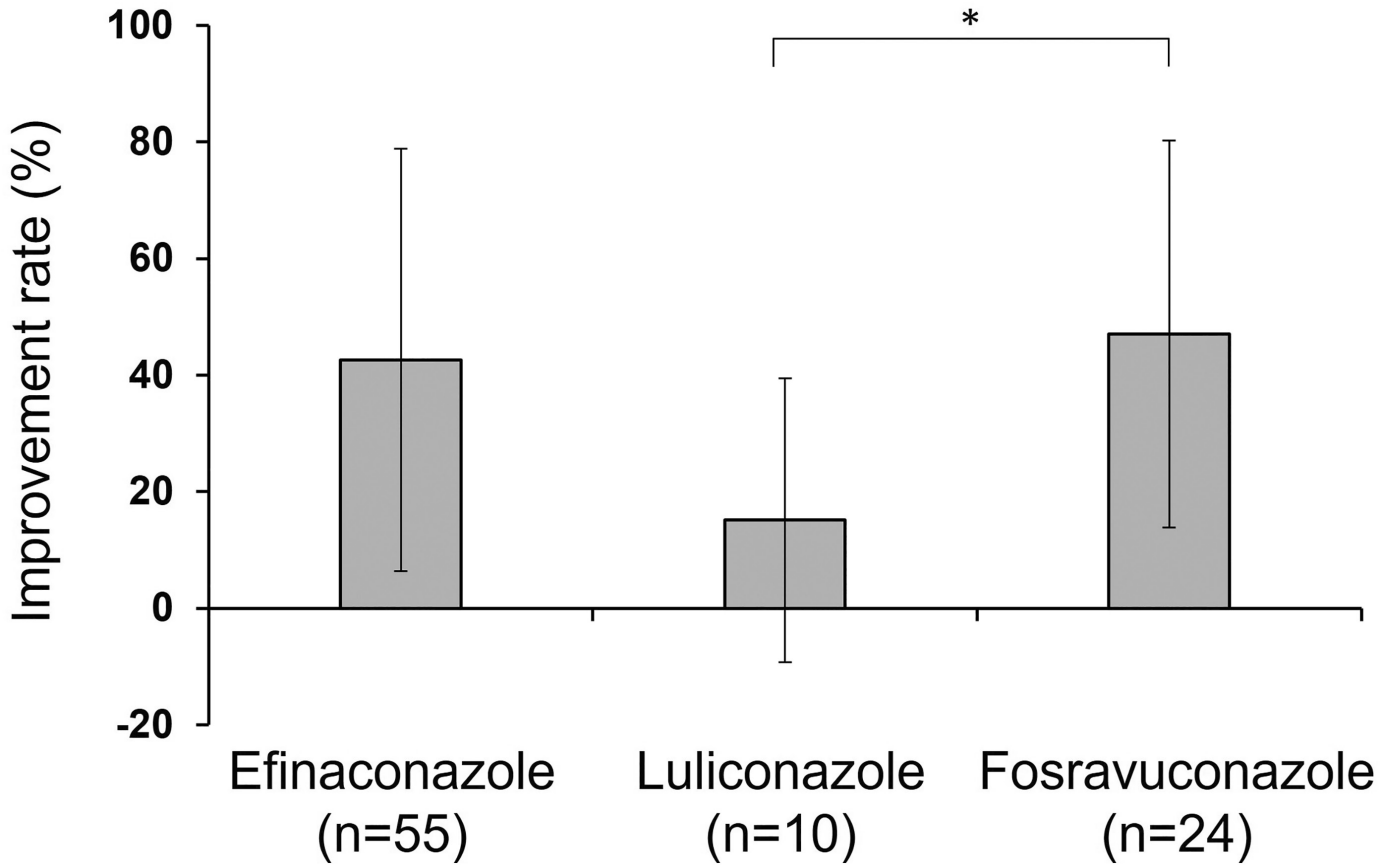
Improvement rates of therapeutic medicines for onychomycosis. Data are presented as mean ± standard deviation. *p* = 0.043 (for all groups by ANOVA), *p < 0.05 (pairwise comparison test [Bonferroni correction]).

**FIGURE 5:**
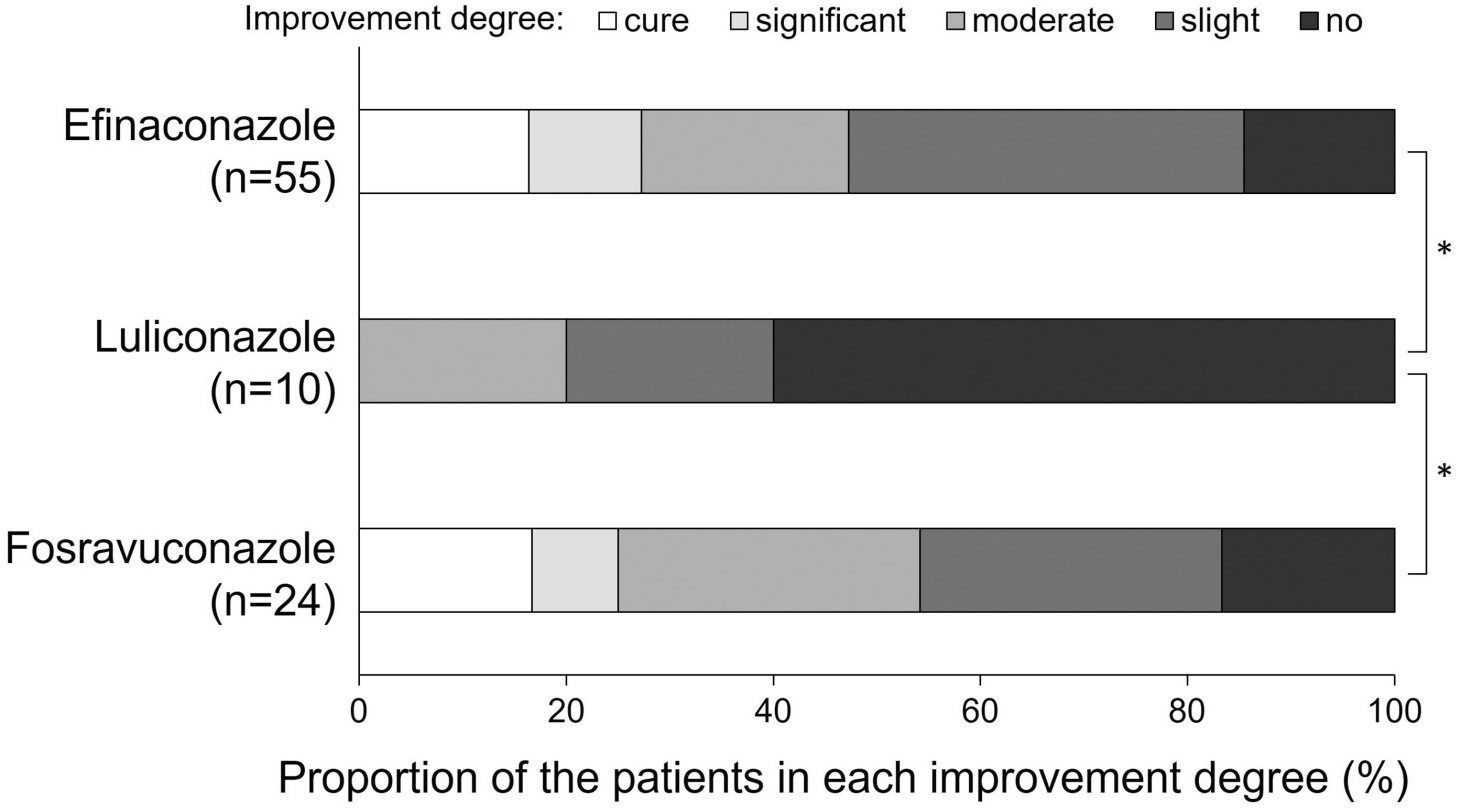
Improvement degrees of therapeutic medicines for onychomycosis. *p* = 0.019 (for all groups by the Kruskal‐Wallis test). *p < 0.05 (Mann–Whitney *U* test [Bonferroni correction]), paired comparison of the distribution of degrees of improvement in each group.

Table 4 shows the affected nail area at the first visit and evaluation (1 year after the first visit), as well as the observation period, improvement rate, and improvement degree, in the efinaconazole‐, luliconazole‐, and fosravuconazole‐treated groups. The affected nail area at the first visit was significantly broader in the fosravuconazole‐treated group than in the efinaconazole‐treated group (*p* = 0.002), and the observation period was significantly shorter in the fosravuconazole‐treated group than in the efinaconazole‐treated group (*p* = 0.007) (Table 4). A significantly higher rate of improvement was observed in the fosravuconazole‐treated group than in the luliconazole‐treated group (*p* = 0.030) (Table 4, Figure 4). Significantly greater degrees of improvement were observed in the efinaconazole‐ and fosravuconazole‐treated groups than in the luliconazole‐treated group (*p* = 0.019 and 0.037, respectively) (Table 4, Figure 5).

**TABLE 4:** Use‐status of therapeutic medicines for onychomycosis and the clinical effects of these medicines.

Evaluation items	Therapeutic medicines for onychomycosis			*p*‐value for all	*p*‐value for		
	1) Efinaconazole	2) Luliconazole	3) Fosravuconazole		1) vs. 2)	1) vs. 3)	2) vs. 3)
Total, *n*	55	10	24				
Affected nail area at first visit							
Mean ± SD	0.46 ± 0.27	0.65 ± 0.30	0.69 ± 0.26	0.002^a^	0.150^b^	0.002^b^	>0.999^b^
Median (IQR)	0.40 [0.23, 0.69]	0.59 [0.43, 0.96]	0.69 [0.47, 1.00]				
Affected nail area at evaluation							
Mean ± SD	0.30 ± 0.26	0.57 ± 0.29	0.40 ± 0.31	0.019^a^	0.016^b^	0.464^b^	0.474^b^
Median (IQR)	0.24 [0.06, 0.50]	0.54 [0.35, 0.87]	0.30 [0.20, 0.65]				
Observation period (week)							
Mean ± SD	50.3 ± 14.8	49.8 ± 12.2	38.9 ± 15.0	0.007^a^	>0.999^b^	0.007^b^	0.152^b^
Median (IQR)	52.1 [44.9, 56.0]	52.0 [45.5, 56.3]	43.5 [22.6, 52.4]				
Improvement rate (%)							
Mean ± SD	42.6 ± 36.2	15.1 ± 24.4	47.1 ± 33.2	0.043^a^	0.074^b^	>0.999^b^	0.030^b^
Median (IQR)	35.2 [16.5, 72.4]	4.7 [0.0, 31.7]	44.9 [18.7, 74.5]				
Improvement degree, *n* (%)				0.019^c^	0.019^d^	>0.999^d^	0.037^d^
No	8 (14.5)	6 (60.0)	4 (16.7)				
Slight	21 (38.2)	2 (20.0)	7 (29.2)				
Moderate	11 (20.0)	2 (20.0)	7 (29.2)				
Significant	6 (10.9)	0 (0.0)	2 (8.3)				
Cure	9 (16.4)	0 (0.0)	4 (16.7)				
Effective improvement	26 (47.3)	2 (20.0)	13 (54.2)	0.191^e^	0.502^f^	>0.999^f^	0.385^f^
(>Moderate)							

Abbreviations: IQR, interquartile range; SD, standard deviation.

^a^ One‐way ANOVA.

^b^ Pairwise comparison test (Bonferroni correction).

^c^ Kruskal‐Wallis test.

^d^ Mann–Whitney *U* test (Bonferroni correction).

^e^ Fisher’s exact test (for all).

^f^ Fisher’s exact test (Bonferroni correction).

## 4 DISCUSSION

The identification of risk factors for the development of tinea pedis and onychomycosis is important to encourage hospital attendance by potential unmedicated patients, which may lead to the prevention of disease progression and the spread of infections to others. In this study, disease risk factors were analyzed using a large‐scale database of the Podiatry Center of Juntendo University Hospital, which includes the data of >1000 patients/year managed in this center for foot diseases. By using this large‐scale database, risk factors could be analyzed with a large amount of sequence data obtained from a single center. This approach enabled consistent diagnostic criteria for diseases such as tinea pedis, onychomycosis, and their identified risk factors.

In this study, data from 337 patients with tinea pedis and 346 patients with onychomycosis were analyzed to identify risk factors. Among the patients with tinea pedis or onychomycosis, 259 patients (~ 75% of each patient population) had both diseases concomitantly. A previous survey conducted with data of 34 730 subjects, obtained from Japanese dermatologists in 2007, demonstrated that 7521 subjects had a tinea pedis, 3474 patients had an onychomycosis, and 2406 subjects had both diseases concomitantly with a concomitant proportion of 32.0%–69.3% of each patient population.^4^ There is a slight discrepancy in the concomitant proportion between this study and the previous survey, and one of the reasons is that previous data were obtained from a questionnaire survey conducted as a multicenter study and included data obtained from unclear or inconsistent diagnostic criteria for the diseases. On the other hand, our study data were obtained from a single‐center database, which included patients managed with consistent diagnostic criteria.

Multivariate logistic regression analysis in this study revealed that advanced age, male sex, diabetes, and LLI were independent risk factors for developing tinea pedis. In addition to these factors, DPN was also an independent risk factor for developing onychomycosis. In previous reports, advanced age and male sex have been identified as risk factors for tinea pedis or onychomycosis.^5^, ^7^ The incidence of these diseases is thought to be high in people of advanced age because of a long period with infection opportunities, weakened immune system, influence of underlying diseases, such as diabetes, reduction in nail growth, and insufficient foot care caused by a decrease in range of motion or bendability. Diabetes is a well‐known risk factor for tinea pedis and onychomycosis.^12^, ^13^, ^14^ Gupta et al. reported that mycological evidence of onychomycosis was present in 26.2% (144/550) of diabetic subjects, and these subjects were 2.77 times more likely to have onychomycosis than normal individuals, according to an international multicenter study.^14^ The prevalence of onychomycosis is thought to be high in patients with diabetes because of the decrease in resistance to onychomycosis infection and poor health management of nails due to circulatory disturbance in the lower limb, poor wound healing, and sensation and vision disorders. It is important to manage onychomycosis properly in patients with diabetes because its risk is increased owing to limb‐threatening infections that could progress to ulcers and amputation, and because of the comorbidities present in diabetics, including DPN, macro‐ and microvascular diseases, and impaired immunity, in addition to foot deformities.^15^, ^16^ LLI is thought to develop into tinea pedis and onychomycosis as a result of a deficiency in the supply of nutrients and oxygen to the lower limbs, thus causing a weakened immune system, poor wound healing in the lower limb, and reduction in nail growth. In the current study, DPN was identified as an independent risk factor for developing onychomycosis but not tinea pedis. The reason for this difference is unclear; however, we speculate that peripheral neuropathy impairs the sensory and algetic perception system in the peripheral region of the body, resulting in an unawareness of *Trichophyton* infection and a more frequent worsening of the infection in the toenail than in other peripheral foot regions.

We also found a statistically significant association between the disease development and the severity of LLI or DPN, evaluated using the SPP measurement or DPNCheckTM, respectively. The current study is the first study to report these findings, and we believe that the LLI test with SPP measurement and the DPN test with DPNCheck™ are useful methods for determining the risk of development of tinea pedis or onychomycosis in clinical practice in Japan.

We investigated the use of therapeutic medicines for onychomycosis, including topical efinaconazole, topical luliconazole, and oral fosravuconazole, which have recently been approved and prescribed in real‐world clinical practice in Japan.^3^ A greater number of patients were treated with efinaconazole than with luliconazole or fosravuconazole (149, 50, and 31 patients, respectively), thus suggesting that efinaconazole is more commonly used for patients with onychomycosis in clinical practice in Japan. Oral fosravuconazole was used in patients with onychomycosis who had a broadly affected nail area at the first visit rather than topical efinaconazole. This result reflects the therapeutic situation in Japan, where oral medicine is used more frequently than topical medicine in patients with severe onychomycosis. In this study, many patients in the analysis dropped out between the initial visit and the 1‐year follow‐up. Although this database study was unable to determine the reasons for dropouts, it is speculated that the reasons may have included cure by the treatment, treatment ineffectiveness, adverse events, and patient circumstances.

The complete cure rates in Japanese patients with onychomycosis are reported to be 28.8% for efinaconazole (11.9% for the vehicle), 14.9% for luliconazole (5.1% for the vehicle), and 59.4% for fosravuconazole (5.8% for the placebo) in each phase III clinical trial.^17^, ^18^, ^19^ Complete cure rates of efinaconazole are also reported as 17.8% and 15.2% (3.3% and 5.5% for the vehicle, respectively) in two international, phase III clinical trials including Japanese and non‐Japanese patients.^20^ The effects of these three drugs cannot be directly compared because there is a difference in the data for the vehicle/placebo, and these efficacy data are obtained from different studies that used different study methods including the different patient backgrounds in race. In our exploratory analysis, the improvement rate of the affected nail area might be higher in patients treated with fosravuconazole than with luliconazole, and it is possible that the improvement degree was higher in patients treated with efinaconazole or fosravuconazole than with luliconazole. The data obtained from this study were the first findings that directly compare the effects of the three drugs; however, there were differences in the affected nail area at the first visit and the observation period between the drugs, and the data were obtained in an exploratory analysis with a small number of patients. Therefore, the difference in the effects of these therapeutic medicines on onychomycosis should be confirmed in a future, well‐designed study with a larger number of patients.

A limitation of this study is that the risk factors were analyzed using the data of patients who visited a podiatry center because of their foot problems. This means that the study population had at least one foot problem and is, therefore, not necessarily consistent with the population in the real‐world setting that includes individuals without foot problems. However, a strength of this database study is that the foot diseases and risk factors, such as LLI and DPN, were diagnosed using unified criteria in a single study site, although, the expertise of the physicians for these diseases could not be unified in this database study.

In conclusion, the risk factors for developing tinea pedis and onychomycosis were analyzed using a large‐scale database. Multivariate logistic regression analysis revealed that advanced age, male sex, diabetes, and LLI were independent risk factors for tinea pedis. In addition to these factors, DPN was found to be an independent risk factor for onychomycosis. We believe that these data are useful for identifying patients who are at high risk of developing tinea pedis and onychomycosis, which can contribute to the discovery of undiagnosed/unmedicated or potential patients with tinea pedis and onychomycosis. This approach may result in disease prevention and suppression in real‐world clinical practice in Japan.

## CONFLICT OF INTEREST STATEMENT

This study was funded by Kaken Pharmaceutical Co., Ltd. Medical writing support was provided by Yoshiaki Kita, Ph.D. of Medical Professional Relations Inc., who was funded by Kaken Pharmaceutical Co., Ltd.
